# Application of a tungsten apron for occupational radiation exposure in nursing care of children with neuroblastoma during ^131^I-meta-iodo-benzyl-guanidine therapy

**DOI:** 10.1038/s41598-021-03843-2

**Published:** 2022-01-07

**Authors:** Yuka Taniguchi, Hiroshi Wakabayashi, Hiroto Yoneyama, Zhuoqing Chen, Kei Morino, Akiko Otosaki, Masako Yamada, Anri Inaki, Daiki Kayano, Seigo Kinuya

**Affiliations:** 1 Division of Nursing, Kanazawa University Hospital, 13-1 Takara-machi, Kanazawa, Ishikawa 920-8641 Japan; 2 Department of Nuclear Medicine, Kanazawa University Hospital, 13-1 Takara-machi, Kanazawa, Ishikawa 920-8641 Japan; 3 Department of Radiological Technology, Kanazawa University Hospital, 13-1 Takara-machi, Kanazawa, Ishikawa 920-8641 Japan

**Keywords:** Paediatric research, Materials for devices, Techniques and instrumentation

## Abstract

The use of effective shielding materials against radiation is important among medical staff in nuclear medicine. Hence, the current study investigated the shielding effects of a commercially available tungsten apron using gamma ray measuring instruments. Further, the occupational radiation exposure of nurses during ^131^I-meta-iodo-benzyl-guanidine (^131^I-MIBG) therapy for children with high-risk neuroblastoma was evaluated. Attachable tungsten shields in commercial tungsten aprons were set on a surface-ray source with ^131^I, which emit gamma rays. The mean shielding rate value was 0.1 ± 0.006 for ^131^I. The shielding effects of tungsten and lead aprons were evaluated using a scintillation detector. The shielding effect rates of lead and tungsten aprons against ^131^I was 6.3% ± 0.3% and 42.1% ± 0.2% at 50 cm; 6.1% ± 0.5% and 43.3% ± 0.3% at 1 m; and 6.4% ± 0.9% and 42.6% ± 0.6% at 2 m, respectively. Next, we assessed the occupational radiation exposure during ^131^I-MIBG therapy (administration dose: 666 MBq/kg, median age: 4 years). The total occupational radiation exposure dose per patient care per ^131^I-MIBG therapy session among nurses was 0.12 ± 0.07 mSv. The average daily radiation exposure dose per patient care among nurses was 0.03 ± 0.03 mSv. Tungsten aprons had efficient shielding effects against gamma rays and would be beneficial to reduce radiation exposures per patient care per ^131^I-MIBG therapy session.

## Introduction

The use of effective shielding materials for protection against radiation is important among medical staff in the field of nuclear medicine. Occupational radiation exposure is not high in nuclear medicine studies; however, it must be reduced by using protective shields while working with radiopharmaceuticals of high activities^1–5^. Metallic materials with a high atomic number and density for shielding against gamma rays are available^6^. In clinical settings, lead and tungsten are used in vial shields, isotope containers, goggles and aprons^2,7,8^. However, common radiation protection aprons are made with a low lead equivalent for shielding against gamma radiation. A greater lead equivalent is required for higher-energy γ-ray emitters such as ^131^I. Tungsten aprons with a higher lead equivalent are commercially available for nuclear facilities. However, they are not used in clinical settings. Aprons with a high shielding capacity can be useful in clinical practice^1–4^.

^131^I-meta-iodo-benzyl-guanidine (^131^I-MIBG) therapy is used for paediatric patients with advanced and refractory neuroblastoma^9^. Paediatric patients must remain in a radiation isolation room alone after the administration of ^131^I-MIBG until external radiation exposures have been reduced to an acceptable level as per national regulations. A previous study reported the occupational radiation doses, with an average total of 0.36 ± 0.18 mSv and a daily dose of 0.07 ± 0.05 mSv/day per patient care, among nurses who use a commercial lead apron^10^. Although the individual radiation exposure was well controlled, radiation exposure risks must be reduced to the lowest level possible^4,11^.

Therefore, the current study first investigated the shielding effects of a commercially available tungsten apron against ^131^I using gamma ray measuring instruments. Further, the occupational radiation exposure among nurses during ^131^I-MIBG therapy for children with high-risk neuroblastoma was evaluated.

## Results

### Evaluation of shielding ability using a gamma camera

**Figure 1:**
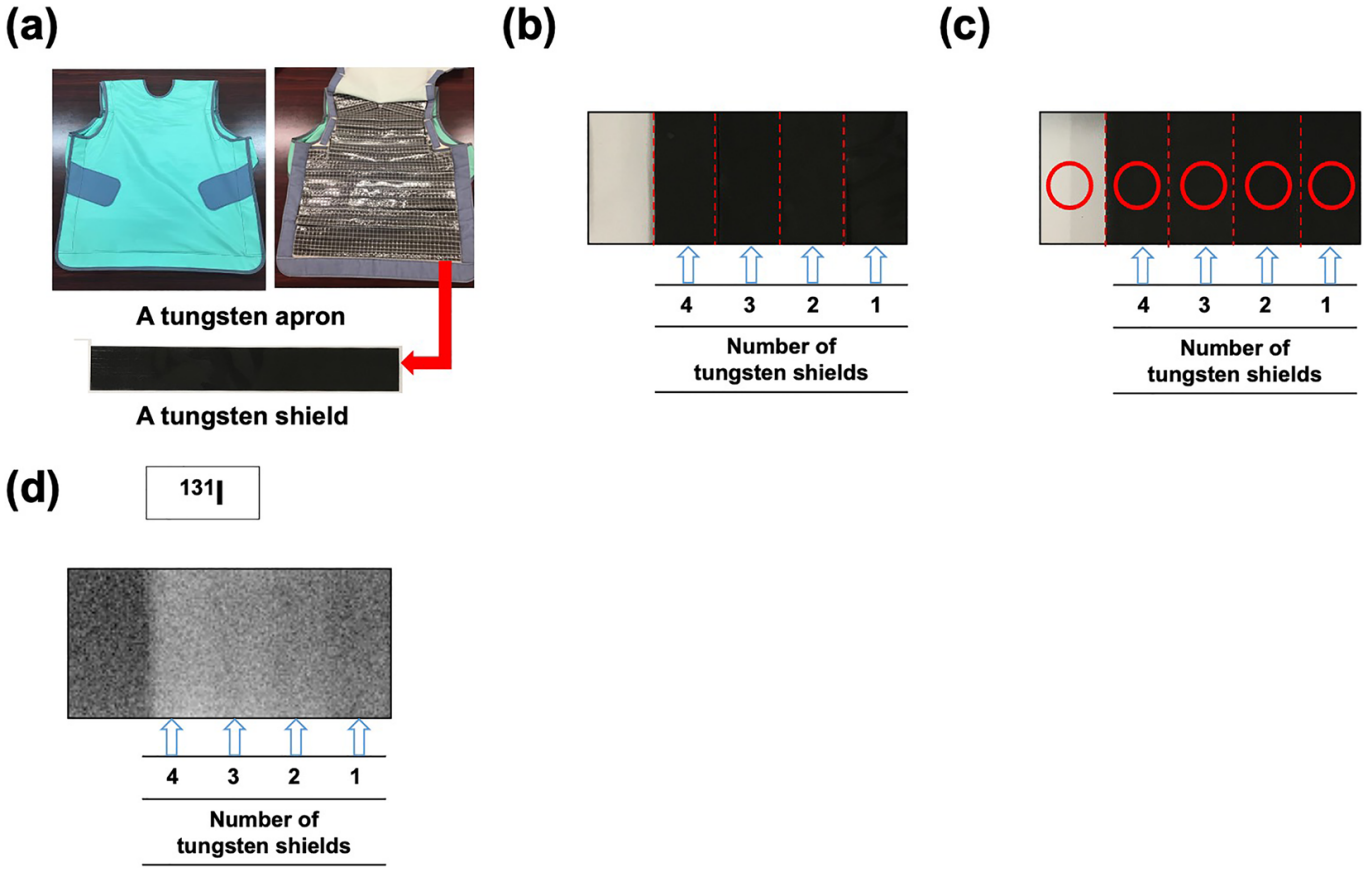
Gamma ray protective effects of a tungsten apron against ^131^I. Attachable tungsten shields (TSs) in a commercial tungsten apron **(a)** were set on the surface-ray source **(b)**. The circular regions of interest (ROIs) (red colour circle) were set in the shielded and non-shielded areas **(c)**. A gamma camera was used for ^131^I imaging **(d)**.

The images of the surface-ray source for ^131^I are presented in Fig. 1. The mean counts per pixel of ^131^I were 71 ± 8 in the non-shielded area, 53 ± 7 in the area shielded with 1 tungsten shield (TS), 46 ± 6 in that shielded with 2 TSs and 38 ± 6 in that shielded with 4 TSs. The mean shielding rate was 0.1 ± 0.006 for ^131^I.

### Evaluation of shielding ability using a NaI scintillator

**Figure 2:**
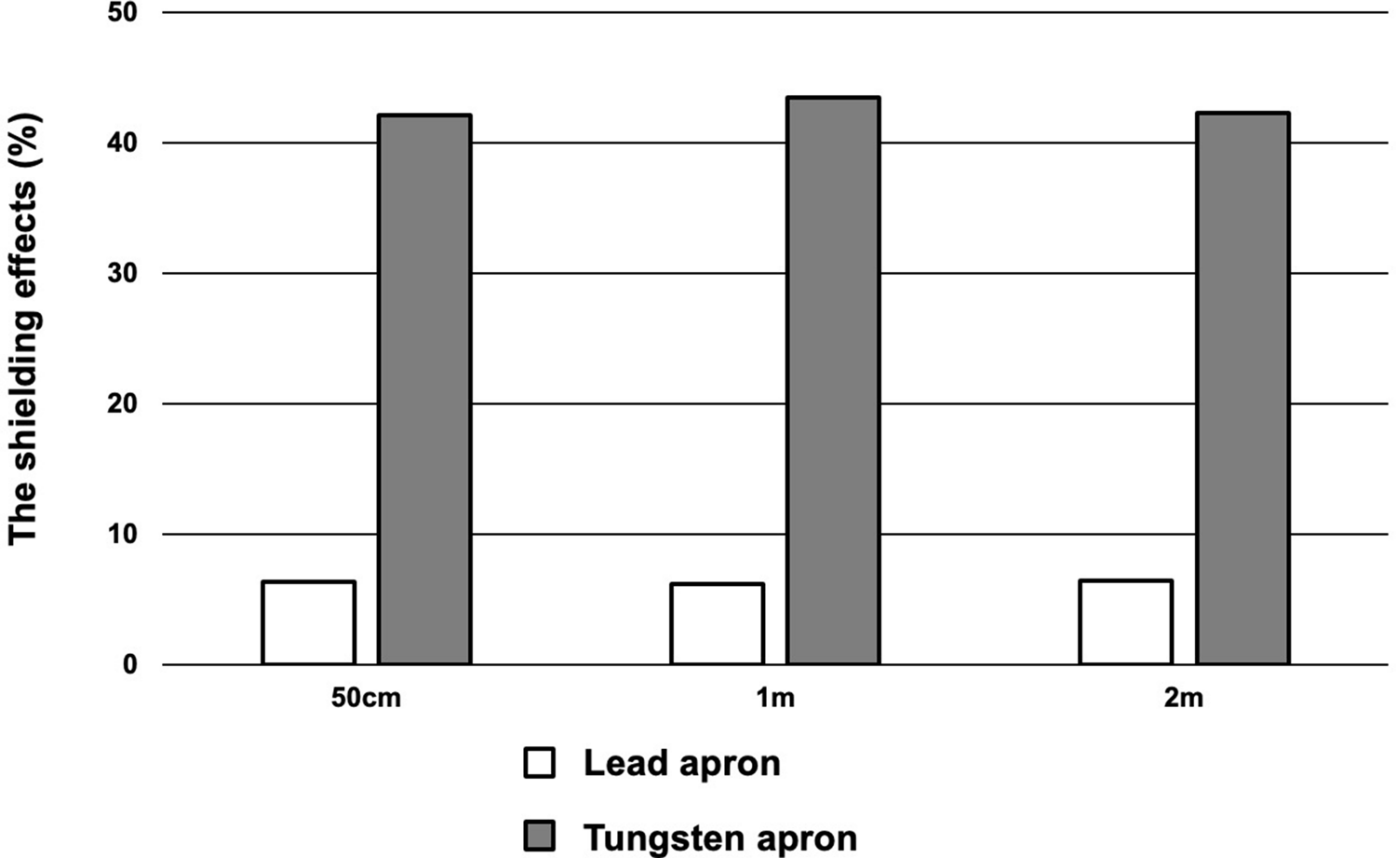
The shielding effects of lead and tungsten aprons against ^131^I. The tungsten apron of 2 mmPb equivalent and the lead apron of 0.35 mmPb equivalent were set at 50 cm, 1 m and 2 m from the radioactive source. The tungsten apron had a significantly higher shielding effect ^131^I at all distances than the lead apron.

The shielding effects of lead and tungsten aprons ^131^I were 6.3% ± 0.3% and 42.1% ± 0.2% at 50 cm, 6.1% ± 0.5% and 43.3% ± 0.3% at 1 m and 6.4% ± 0.9% and 42.6% ± 0.6% at 2 m, respectively (Fig. 2). The tungsten apron had a significantly higher shielding effect ^131^I at all distances than the lead apron (*p* < 0.0001)*.*

### Occupational radiation exposure

Patients aged ≤ 12 years who presented with neuroblastoma received seven sessions of ^131^I-MIBG therapy (666 MBq/kg, median body weight: 17 kg, range 10–38 kg). They were four women and three men (median age at ^131^I-MIBG therapy: 4 years, range 1–12 years). Patients stayed in the isolation room for a mean duration of 4 ± 1 (range 3–5) days. The average number of nurses who worked per day during the patients’ stay in the isolation room to reduce radiation exposure was 3 ± 1 (range 2–7). The self-care score was 16 ± 6 (range 10–24).

**Figure 3:**
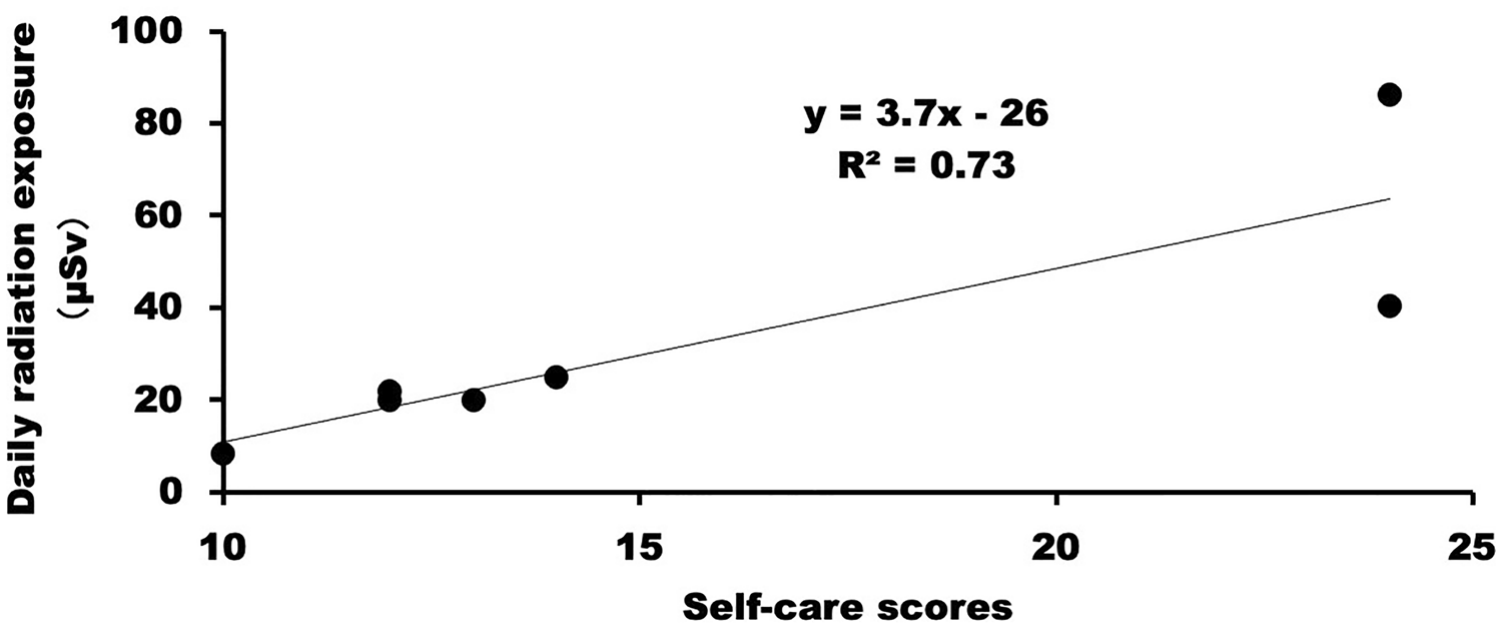
Relationship between self-care score and daily radiation exposure. The self-care scores were positively correlated with daily radiation exposure among nurses.

The total radiation exposure among nurses per patient care per ^131^I-MIBG therapy session was 0.12 ± 0.07 (range 0.04–0.26) mSv. The average daily radiation exposure among nurses per patient care was 0.03 ± 0.03 (range 0.001–0.06) mSv. None of the nurses had a radiation exposure dose of > 0.1 mSv/day after ^131^I-MIBG therapy. The mean daily percentages of the total radiation exposure dose after days 0, 1, 2 and 3 were 33% ± 13%, 28% ± 10%, 24% ± 14% and 11% ± 5%, respectively (Tukey’s HSD test: day 0 vs. 2 and day 0 vs. 3, *p* < 0.05). The self-care scores were positively correlated with daily radiation exposure (horizontal axis: score, vertical axis: μSv, y = 3.7x − 26; squared correlation coefficient = 0.73, *p* < 0.05, Fig. 3) among nurses.

## Methods

### Ethical considerations and study registration

This study was conducted in accordance with the Declaration of Helsinki and the International Committee for Harmonization of Good Clinical Practice guidelines. Moreover, it was approved by the clinical ethics committee of Kanazawa University (#20-3353: approved date 06/27/2020). An opt-out method was used to assess personal data on occupational radiation exposure in this research. Participants can withdraw from the study within a specified period. The Institutional Review Board of Kanazawa University Hospital approved a clinical trial on ^131^I-MIBG therapy for paediatric patients with high-risk neuroblastoma. Written informed consents were obtained from all participants or their parents or guardians prior to commencing ^131^I-MIBG therapy.

### Evaluation of shielding ability using a gamma camera

Attachable TSs in a commercial tungsten apron (thickness of TS 1 mm ≈ 1 mm Pb equivalent; Chiyoda Technol Corp., Japan) (Fig. 1a) were set on the surface-ray source (GPA-09A, Toshiba, Japan) (Fig. 1b). The resin-based TS of the tungsten apron used in this study has a specific gravity of approximately 11.34 g/cm^3^. ^131^I (37 MBq) was used as a radioactive source. A gamma camera (Symbia T6, Siemens Medical Solutions USA Inc., Hoffman Estates, IL, USA) with high-energy general-purpose collimators was used for ^131^I imaging (energy window: 364 keV ± 7.5%). The acquisition time was 30 min for ^131^I. The pixel size was 2.4 mm, and the circular regions of interest (ROIs) (red colour circle as shown in Fig. 1b) were set in the shielded and non-shielded areas. The ROI setting was repeated three times. The mean count density per pixel (mean count) of each ROI was analysed using an e-soft workstation (Siemens Medical Solutions USA Inc., Hoffman Estates, IL, USA). The shielding rate of the tungsten shield was calculated using the rate of attenuation and the following formula: ${\text{S}}hielding \;rate = {\text{LN }}\left[ {{\text{inverse}}\;{\text{number}}\;{\text{of }}\;{\text{reduction}}\;{\text{rate}}\;{\text{by }}\;{\text{the}}\;{\text{ shields}}} \right] \div {\text{thickness }}\;{\text{of }}\;{\text{TSs}}\;{ }\left[ {{\text{mm}}} \right]$.

### Evaluation of shielding ability using a NaI scintillator

The tungsten apron of 2 mmPb equivalent and the lead apron of 0.35 mmPb equivalent were set at a distance of 50 cm, 1 m and 2 m from the radioactive source (^131^I [370 MBq]). The thyroid uptake measurement systems (AZ-800-HS, Anzai Medical Co., Ltd., Tokyo, Japan), which had a mono-photomultiplier tube and 2-inch (diameter) × 2-inch (thickness) NaI crystal, was used for ^131^I (energy window: 364 keV ± 10%) counting rate. The radioactive source was surrounded by lead blocks except in the scintillator’s direction and was placed at the same height as the scintillator. The apron was placed in front of the detector. The acquisition time was 1 min for ^131^I (370 MBq), and the measurements were repeated five times. After time and background correction, the average value was calculated. Next, we evaluated the shielding effect by calculating the transmittance of lead or tungsten apron using the following formula: $Shielding \;effect = \left( {1 - measured\; values \;with\; an\; apron \div measured \;values\; without \;an \;apron} \right) \times 100.$

### Occupational radiation dose evaluation

This study included 18 trained nurses who worked in our hospital and who cared for patients aged ≤ 12 years who had neuroblastoma and who were administered ^131^I-MIBG therapy. Nurses routinely used a pocket dosimeter (MYDOSE mini PDM-222C, Hitachi Aloka Medical, Tokyo, Japan) on the lower abdomen among women and on the chest among men under the tungsten apron in the isolation ward. Next, the cumulative exposure dose was recorded. Nurses had free access to patients. When paediatric patients request nursing care, including feeding, watching videos and reading books, the family member in another room equipped with a monitoring system conveyed such concerns to the nurses. The number of nurses caring for a patient each day was counted. The radiation exposure dose was expressed in μSv units. The total radiation exposure was the sum of radiation exposures of all nurses involved in administering the ^131^I-MIBG therapy. The average daily radiation exposure was calculated as the ratio of total radiation exposure per patient care to the length of patient stay (days) in the isolation room. The daily percentage of total radiation exposure was calculated using the following formula: $Daily \;percentage\; of\; total \;radiation \;exposure = daily\; radiation \;exposure \div total\; radiation\; dose \times 100.$ ^131^I-MIBG was intravenously administered within 60 min from the start time (approximately 14:00).

### General radiation protection of nurses

The medical staff was educated about radiation protection and ^131^I-MIBG therapy annually. Then, nurses wore the tungsten apron, radiation shield and gloves, and their distance from patients was ensured to maximum as much as possible. Based on Japanese regulations about occupational radiation exposure, female and pregnant staff should not be exposed to a dose of ≥ 5 mSv every 3 months and a dose of 2 mSv on the abdomen during the gestation period, respectively. This method is based on the International Commission on Radiological Protection (ICRP) Publication 60 (1990 recommendations of ICRP)^12^. The person in charge must adopt measures to reduce radiation exposure if the worker is exposed to higher levels than recommended in the guidelines. The radiation exposure doses among trained nurses working in nuclear medicine facilities must not exceed 0.10 mSv/day and 0.30 mSv/month, and the doses of radiation exposure were monitored using the pocket dosimeter.

### ^131^I-MIBG therapy

^131^I-MIBG therapy was planned based on draft guidelines on the appropriate use of ^131^I-MIBG radiotherapy for neuroendocrine tumours based on the Japanese Society of Nuclear Medicine and the European Association of Nuclear Medicine (EANM) for procedure guidelines^13,14^. The inclusion and exclusion criteria were based on the jRCTs041180030 (https://jrct.niph.go.jp/en-latest-detail/jRCTs041180030)^15^ and jRCTs041180041 (https://jrct.niph.go.jp/en-latest-detail/jRCTs041180041) clinical trials. In the radiation isolation room, all paediatric patients received ^131^I-MIBG therapy (POLATOM, Otwock, Poland). Nurses cared for children who had incontinence and those who needed help in changing clothes and bedsheets, laying polyethylene filter paper on the floor and discarding diapers if needed. Patients could exit the isolated room when the radiation dose was ≤ 30 μSv/h or when the body’s remaining radiation dose was ≤ 499.5 MBq.

### Self-care score

We used our self-care score to determine independence by evaluating the level of assistance required to perform necessary daily activities in an isolated room^10^. It comprises 10 items categorised into two dimensions: daily activities including 9 items and recognition of the treatment including one item. The daily activities included the following items: (1) eating food, (2) taking internal medicines, (3) performing urination and defecation, (4) wearing and removing the diaper, (5) handling urinary incontinence, (6) taking a shower, (7) wearing and removing clothes, (8) using a monitoring system and (9) sleeping by themselves. Each item was measured on a 3-point rating scale: no assistance, partial assistance and full assistance (scores of 1, 2 and 3, respectively). The recognition of the treatment was measured on a 2-point rating scale of understanding (score 1) or not understanding (score 2). The total score ranges from 10 to 29. Medical records were collected and scored by the trained nurses retrospectively.

### Statistical analysis

Data were expressed as mean ± standard deviation unless stated otherwise. Statistical analyses were performed using the JMP software (version 14, SAS Institute Inc., Cary, NC, USA). Matched pair analyses were performed to compare differences in the shielding effect of lead and tungsten apron. The correlation between the self-care score and daily radiation exposure doses among nurses was compared via a linear regression analysis. To compare the percentage of radiation exposure doses per the total radiation dose, analyses were performed using the analysis of variance and the Tukey’s honest significant difference (HSD) test. A *p-value* of < 0.05 was considered statistically significant.

## Discussion

This study firstly reported about the use of tungsten aprons in clinical practice. This material had a significant shielding effect against ^131^I, and none of the nurses exceeded the hospital’s recommended exposure dose (0.1 mSv/day) per worker during ^131^I-MIBG therapy in children with neuroblastoma. The occupational radiation exposure dose was within the normal range, and the tungsten apron was considered effective against occupational radiation exposure.

The shielding efficacy test showed the use of a tungsten apron for protection against ^131^I. The resin-based tungsten sheet of the tungsten apron used in this study has a specific gravity of approximately 11.34 g/cm^3^, the same as a lead. Because the density and the atomic number of the absorbing medium determine the attenuation coefficient^6^, even tungsten-based products with an atomic number of 74, which is lower than that of lead, i.e., 82, can provide the same level of radiation protection as a lead-based product. The efficacy of a tungsten apron for protection against occupational radiation was higher than that of a lead apron based on previous studies^16–22^. When using commercial lead aprons, the total occupational radiation exposure dose among nurses per patient care per ^131^I-MIBG therapy session was 0.36 ± 0.18 mSv, and the average daily radiation exposure dose among nurses per patient care was 0.07 ± 0.04 mSv^10^. In this study, the total radiation exposure dose among nurses per patient care per ^131^I-MIBG therapy session was 0.12 ± 0.07 mSv, and the average daily radiation exposure dose among nurses per patient care was 0.03 ± 0.03 mSv. In total, 18 nurses who used the lead apron had an exposure dose of > 0.10 mSv/day in a previous report. However, in this study, the dose among all nurses who used the tungsten apron was below 0.10 mSv/day. The occupational radiation exposure dose using the tungsten apron was reduced to approximately half to one-third of that with the lead apron, which was less effective than expected based on the shielding effect test. Even with the use of the tungsten apron, the results might have been affected by basic radiation protective factors such as exposure time and distance from the child. Radiation exposure can be accumulated over time with prolonged exposure, and a greater distance from the radiation source can reduce radiation exposure^23–25^.

In this survey, the maximum total radiation dose per treatment session of a child aged 1 year old was 0.26 mSv. We believe that this value was within acceptable limits considering that it was obtained without a family caregiver. The exposure dose among family caregivers during ^131^I-MIBG therapy ranged from 0 to 8.99 mSv^16–22^. The radiation doses of some caregivers of younger patients were > 5.0 mSv. On the other hand, in the case of ^131^I therapy for thyroid diseases, the most famous nuclear medicine treatment, a medical worker’s annual occupational radiation exposure is < 2.2 mSv^25,26^. The occupational dose of up to 0.26 mSv per ^131^I-MIBG therapy session in this study is considered to be higher than that of ^131^I treatment. The care strategies in the isolation room differ per country, and trained nurses provide care to paediatric patients instead of family caregivers in our hospital. Isolation is a situation in which the child is likely to experience fear, anxiety and stress. These feelings are intensified when the child is placed in an unfamiliar environment, such as an isolation ward. If occupational radiation exposure is well controlled with tungsten aprons, it would reduce nurses’ anxiety about occupational radiation exposure.

However, the commercially available tungsten apron is expensive and heavy, and these can be an area for improvement. Our tungsten apron of 2 mmPb equivalent weighs approximately 10 kg. Because we do not permit family members to care for paediatric patients in the isolation room, nurses must provide nursing care during treatment. Further, nurses will experience physical burdens caused by prolonged wearing of the tungsten apron during care. Although our results showed that it is important to use a tungsten apron to lower radiation exposure dose, we should consider its disadvantages in clinical settings.

The current study has several limitations. First, the number of participants was extremely small. Second, we could not consider the distance from the patient and time spent in an isolation room. Therefore, to improve clinical experience and nursing care for children, the use of a tungsten apron must be further examined in future clinical settings.

## Conclusion

The tungsten apron was an effective shielding material against gamma ray. Occupational radiation exposure was well controlled among nurses who used the tungsten apron during ^131^I-MIBG therapy for children with high-risk neuroblastoma. Hence, it is a promising shielding material against occupational radiation exposure among medical staff in the field of nuclear medicine.
